# Connexin 26 is Down-Regulated by KDM5B in the Progression of Bladder Cancer

**DOI:** 10.3390/ijms14047866

**Published:** 2013-04-11

**Authors:** Xin Li, Yongping Su, Jinhong Pan, Zhansong Zhou, Bo Song, Enqing Xiong, Zhiwen Chen

**Affiliations:** 1 Urologic Institute of PLA, Southwest Hospital, Third Military Medical University, Chongqing 400038, China; E-Mails: xinlicq@yahoo.com (X.L.); zhansongzhoucq@yahoo.com (Z.Z.); bosongcq@yahoo.com (B.S.); enqingxiongcq@yahoo.com (E.X.); zhiwenchencq@yahoo.com (Z.C.); 2 State Key Laboratory of Trauma, Burns and Combined Injury, Institute of Combined Injury, Third Military Medical University, Chongqing 400038, China

**Keywords:** KDM5B, connexin 26, HT1376 human bladder cancer cells, T24 human bladder cancer cells, real-time qPCR, immunohistochemistry, spearman’s rank correlation

## Abstract

Connexin 26 (Cx26) expression is down-regulated and KDM5B (H3K4 demethylase) is up-regulated in the progression of bladder cancer, suggesting that Cx26 expression may be down-regulated by KDM5B in bladder cancer. To test the hypothesis, the HT1376 and T24 human bladder carcinoma cells were transfected with the plasmids pcDNA3.1-KDM5B, and caused the down-regulation of Cx26 expression. In contrast, the HT1376 and T24 cells transfected with the plasmids pTZU6+1-shRNA-KDM5B1 and pTZU6+1-shRNA-KDM5B2 caused the up-regulation of Cx26 expression. Immunohistochemistry and Spearman’s rank correlation analysis showed that the immunohistochemical expression of KDM5B and Cx26 was inversely related in bladder carcinoma tissues but no relationship in benign tissues. Taken together, these results indicate that KDM5B represses Cx26 expression in the bladder cancer development. Thus, a negative value to Cx26 immunohistochemical expression and a positive value to KDM5B immunohistochemical expression could be an ancillary diagnosis of primary bladder malignancy.

## 1. Introduction

The worldwide estimate for new cases of bladder cancer is 261,000 annually [1], while the causes of bladder cancer remain unknown in most cases [2]. Histone demethylase has been suggested to be associated with various cancers [3–5]. For instance, KDM5B (also named JARID1B) is an H3K4me3/me2/me1 specific lysine demethylase belonging to the JmjC domain-containing family of histone demethylases (JHDMs) [6,7]. KDM5B expression is significantly up-regulated in bladder cancer, acute myelogenous leukemia, breast cancer, chronic myelogenous leukemia, cervical cancer and renal cell carcinoma compared with corresponding non-neoplastic tissues, indicating its involvement in many types of human cancer [8]. Quantitative RT-PCR analysis confirms that expression levels of KDM5B are significantly higher in human bladder cancer tissues than in their corresponding non-neoplastic bladder tissues (*p* < 0.0001) [8]. The expression profile analysis of clinical tissues also reveals up-regulation of KDM5B in various kinds of malignancies. Transfection of KDM5B-specific siRNA into various bladder cancer cell lines significantly suppresses the proliferation of cancer cells and increased the number of cells in sub-G1 phase [8]. KDM5B is widely expressed in breast cancer cell lines. Down-regulation of expression of KDM5B using shRNAi in the breast cancer cell line MCF-7 cells result in a dramatic decrease in E2 stimulated tumor growth in nude mice [9].

In contrast, connexins make up a gene family encoding proteins that form intercellular channels known as gap junctions, which is the most important for the direct communication between adjacent cells and allows exchange of ions, second messengers, small metabolites, and peptides for basic cell physiological activities [10]. Signals of contact inhibition, apoptosis, differentiation, and localization are transferred from adjacent cells through gap junctions to maintain cellular homeostasis while uncontrollable proliferation and poor differentiation will increase the risk of cancers [11]. The dysfunction of connexins may affect cell proliferation, differentiation, and localization, which may be correlated with tumorigenesis [12]. Decreases in connexin expression and loss of intercellular communication have been associated with the malignant phenotype in some animal and human cells [13] while enhancement of connexins function has a profound effect for growth inhibition of cancers [14,15]. Aberrant expression and down-regulation of connexin 26 (Cx26) are related with the progression of some cancers [16–18]. In breast cancer, it is demonstrated that down-regulated expression of Cx26, leading to the lack of gap junctional intercellular communication (GJIC), is a molecular event [19], which may reduce gap junction signaling. Among the connexins family, Cx26 are widely reported to be inversely related with bladder cancer [20–22]. Increased confluence of the cultured normal human urothelial cells is associated with up-regulation of Cx26. Cx26 expression is decreased in the bladder cancer cells. These data suggest that alterations in the regulation of Cx26 expression may contribute to the malignant phenotype in bladder cancer [13,20]. Furthermore, transfection of Cx26 can significantly inhibit the growth of human bladder carcinoma [21].

Recent studies have provided evidence for a diverse role of histone demethylase in the expression of various genes [23–25]. Thus, Cx26 expression may be regulated by histone demethylase. From the inverse expression pattern of Cx26 and KDM5B in bladder neoplasm [8,13,20], Cx26 expression may be down-regulated by KDM5B in the progression of bladder cancer. To test the hypothesis, the expression of Cx26 and KDM5B were investigated under different situations.

## 2. Results and Discussion

### 2.1. The Expression Levels of Cx26 Were Inhibited by KDM5B

**Figure 1:**
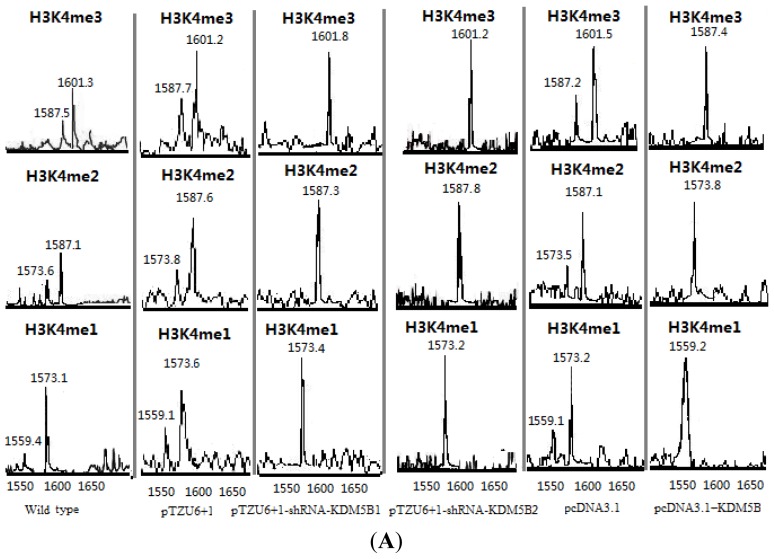

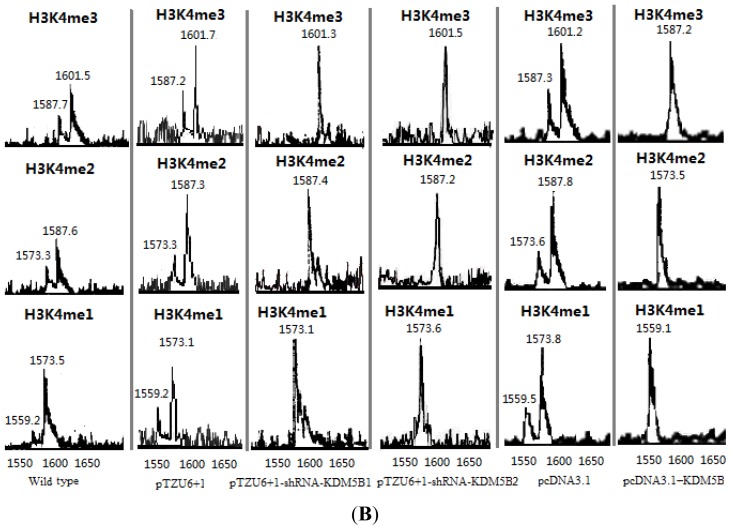
The demethylating bioactivity of KDM5B in the non-transfected and transfected HT1376 and T24 cells. (**A**) The demethylating bioactivity of KDM5B in the non-transfected and transfected HT1376 cells; (**B**) The demethylating bioactivity of KDM5B in the non-transfected and transfected T24 cells. Each panel contains spectrum for either mono- (me1), di- (me2), and trimethylated (me3) 15-aa peptides near N-terminus of histone 3 (H3K4Me3/2/1: ART(Me3/2/1)KQTARKSTGGKA; the molecular weights are 1601,1587 and 1573 Da for H3K4Me3/2/1 respectively) incubated with the non-transfected and transfected HT1376 and T24 cells extracts. The appearance of a peak corresponding to demethylated peptide. The shift corresponds to a loss of 14 Da because –CH_3_ is removed and a –H is added.

The low bioactivity of KDM5B demethylase could be detected in HT1376 and T24 bladder invasive transitional cell cancer cell lines and the cell lines transfected with pcDNA3.1 and pTZU6+1. KDM5B was able to remove tri-, di- and monomethyl groups from methylated H3K4 (Figure 1). The non-transfected and transfected HT1376 and T24 cell lines with pCDNA3.1 and pTZU6+1 had the similar demethylating activities. On the other hand, the HT1376 and T24 cells transfecting with pTZU6+1-shRNA-KDM5B1 and pTZU6+1-shRNA-KDM5B2 could efficiently inhibit KDM5B activity (Figure 1). Conversely, the HT1376 and T24 cells were transfected with pcDNA3.1-KDM5B, showing the high bioactivity of demethylase (Figure 1).

**Figure 2:**
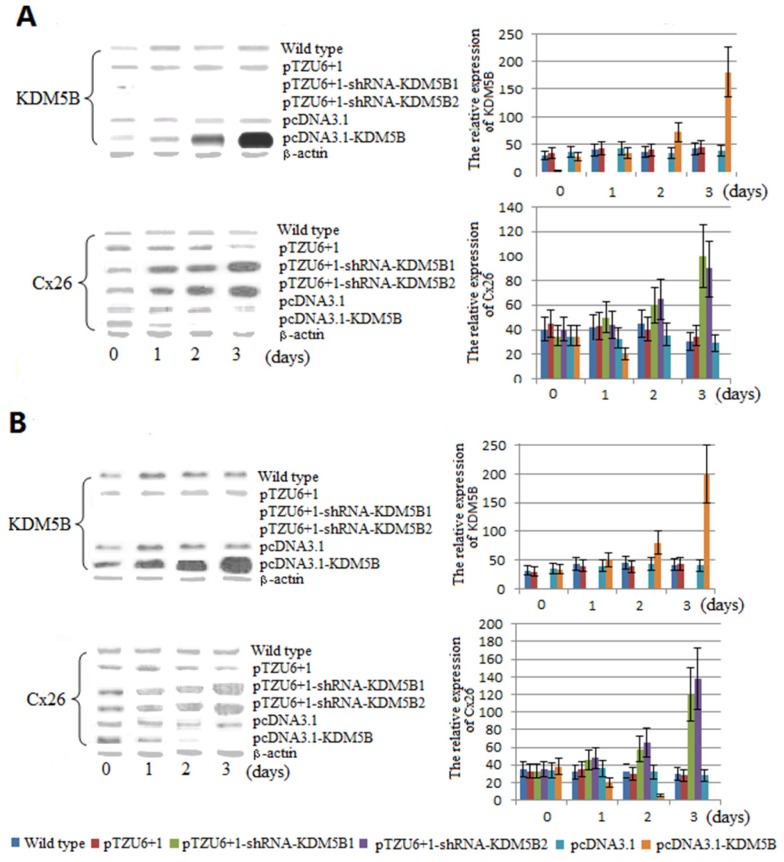
The relative expression of Cx26 and KDM5B in the non-transfected and transfected HT1376 and T24 cells. (**A**) The relative expression of Cx26 and KDM5B in the non-transfected and transfected T24 cells; (**B**) The relative expression of Cx26 and KDM5B in the non-transfected and transfected HT1376 cells. Left panel, western blots analysis for the expression of KDM5B and Cx26. β-actin was shown as a control. Right panel, Real-time qPCR analysis for the relative expression of Cx26 and KDM5B. Each bar represented the mean ± S.D. of three independent experiments.

Western blots analysis showed that the expression of Cx26 and KDM5B was stable in the non-transfected and transfected HT1376 and T24 cells transfected with pcDNA3.1 and pTZU6+1 (Figure 2, left panel). In the HT1376 and T24 cells transfected with pcDNA3.1-KDM5B, the expression of Cx26 was reduced and KDM5B was increased during three-day culture. On the third day, Cx26 was inhibited completely in the HT1376 and T24 cells while the expression level of KDM5B reached the highest level (Figure 2, left panel). Meanwhile, the growth rate of the transfected HT1376 and T24 cells was enhanced by 11% and 16% respectively comparing with corresponding non-transfected cell lines (Supplemental Figure 1A). Conversely, in the HT1376 and T24 cells transfected with pTZU6+1-shRNA-KDM5B1 and pTZU6+1-shRNA-KDM5B2, the expression of Cx26 was increased and KDM5B could not be observed during three-day culture (Figure 2, left panel), while the growth rate of the transfected HT1376 and T24 cells were reduced by 14% and 23% comparing with corresponding non-transfected cell lines (Supplemental Figure 1B).

Real-time qPCR analysis showed that Cx26 expression level in the HT1376 and T24 cells transfected with pcDNA3.1-KDM5B was the lowest in all groups (Figure 2, right panel). Conversely, Cx26 expression level was the highest in the HT1376 and T24 cells transfected with pTZU6+1-shRNA-KDM5B1 and pTZU6+1-shRNA-KDM5B2 in all groups (Figure 2, right panel). All the results suggested that Cx26 expression could be strictly inhibited by KDM5B.

Histones constitute the basic scaffold proteins around which DNA is wound to form the highly ordered structure of chromatin. Histones, and in particular their tails, are subjected to a plethora of post-translational modifications that have been implicated in chromatin remodeling and closely linked to transcriptional regulation, DNA replication, and DNA repair [26,27]. Histone methylation represents the most common modifications of the histone tails. The effect of histone methylation impacts on the transcriptional activity of the underlying DNA by acting as a recognition template for effector proteins modifying the chromatin environment and leading to either repression or activation. Thus, histone demethylase can be associated with either activation or repression of transcription depending on which effector protein is being recruited [12]. From the inverse expression pattern between Cx26 and KDM5B, Connexin 26 may be down-regulated by KDM5B in the progression of bladder cancer.

### 2.2. The Expression of KDM5B is Up-regulated and Cx26 is Down-Regulated in the Progression of Bladder Cancer

**Figure 3:**
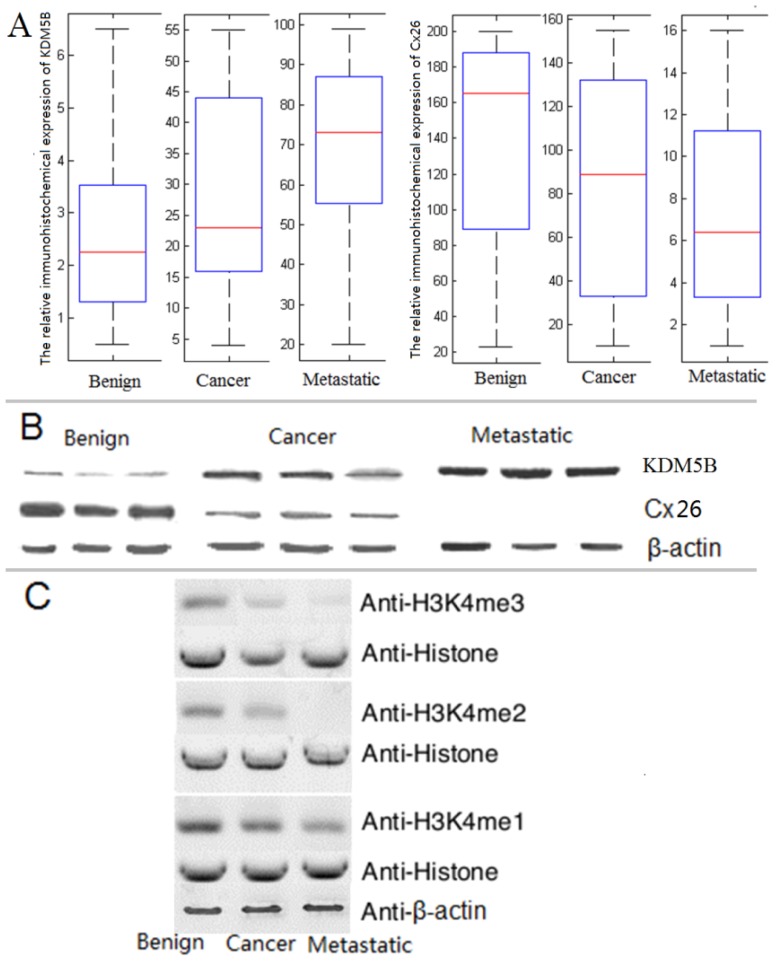
Expression of Cx26 and KDM5B in normal bladder, normal bladder cancer and metastatic bladder cancer. (**A**) The relative immunohistochemical expression of Cx26 and KDM5B in normal bladder, normal bladder cancer and metastatic bladder cancer. The red bars in the boxes were average activities and the boxes represented 95% of the samples. The error bars were above or below the boxes. The patient numbers for each category were: *n* = 38 for benign bladder, *n* = 46 for normal bladder cancer, and *n* = 55 for metastatic bladder cancer; (**B**) Western blot analysis of expression of Cx26 and KDM5B in normal bladder, normal bladder cancer and metastatic bladder cancer. β-actin was shown as a control. Each sample was tested in triplicate; (**C**) The demethylating activities of KDM5B in normal bladder, normal bladder cancer and metastatic bladder cancer. β-actin was shown as a control.

Considering the different clinic situations of bladder cancer, the immunohistochemical expression of Cx26 and KDM5B was measured in normal bladder (marked as “benign”, no tumor was found in the bladder), normal bladder cancer (marked as “cancer”, the tumor was found on or under the inner lining of the bladder; the tumor grew into the muscle layer of the bladder) and metastatic bladder cancer (marked as “metastatic”, the tumor grew through the bladder muscle into the fat layer surrounding the bladder; the tumor spread to surrounding organs, such as the prostate, bowel, vagina, or uterus). The immunocytochemistry analysis showed that the immunohistochemical expression of KDM5B was increased from normal bladder to bladder cancer. The expression of KDM5B reached the highest level in metastatic bladder cancer (Figure 3A). The results suggested that the expression of KDM5B was up-regulated in the development of bladder cancer, which was consistent with previous report [8]. Conversely, the immunohistochemical expression of Cx26 was reduced from normal bladder to bladder cancer. The expression of Cx26 reached the lowest level in metastatic bladder cancer (Figure 3A). The data implied that the reduced expression of Cx26 could promote the growth of bladder cancer, which was also accordant with previous report that the decreased Cx26 expression was observed in bladder cancer [20]. The Cx26 gene encodes a protein involved in GJIC and is a putative tumor suppressor [21]. The reduced expression of Cx26 will affect the formation of GJIC. The homeostatic control is mediated by GJIC in normal cells, while cancer cells lack GJIC [28]. Thus, the decreased Cx26 expression will promote the progression of bladder cancer.

The western blots analysis showed that the expression of KDM5B reached the highest level while Cx26 expression could not be observed at the advanced stage of bladder cancer (Figure 3B). The changing trend of KDM5B expression was the inverse of that of Cx26, suggesting that Cx26 might be strictly controlled by KDM5B.

To make sure the bioactivity of KDM5B, the demethylating characters of the enzyme were reexamined. KDM5B was able to remove tri-, di- and monomethyl groups from methylated H3K4. The activity was increased from normal bladder to bladder cancer (Figure 3C). The changing trend was corresponding to the trend showed in western blot analysis (Figure 3B).

### 2.3. The Stringent Converse Relationship between Cx26 and KDM5B

**Figure 4:**
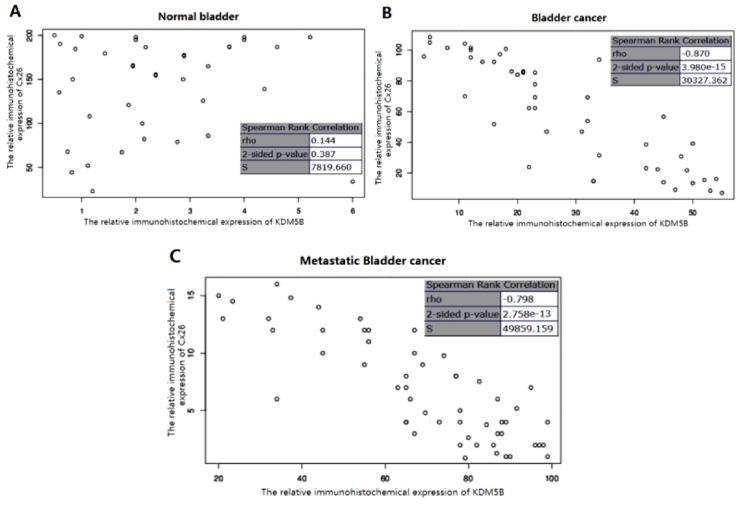
The relative immunohistochemical expression relationship between the Cx26 and KDM5B. Statistical analysis was done by Spearman’s rank correlation test. The value of rho falls between −1 and −0.5, there is a strong negative correlation. The value falls between 0 and 0.5, there is a weak positive correlation. (**A**) The relative immunohistochemical expression relationship between the Cx26 and KDM5B in normal bladder; (**B**) The relative immunohistochemical expression relationship between the Cx26 and KDM5B in normal bladder cancer; (**C**) The relative immunohistochemical expression relationship between the Cx26 and KDM5B in metastatic bladder cancer.

The Spearman’s rank correlation coefficient showed that no relationship between the Cx26 and KDM5B expression was observed in normal bladder (*p* > 0.05) (Figure 4A) while the two variables were inversely related in bladder cancer (*p* < 0.001) (Figure 4B,C). The stringent relation between the Cx26 and KDM5B expression could not be found in benign tissues, which might be caused by the too low expression of KDM5B in normal bladder. The inverse relation between the two variables suggested that Cx26 expression was down-regulated by KDM5B *in vivo* in the progression of bladder cancer.

Bladder cancer is the most common urological cancer. Pathological diagnosis is a key factor for correct and on-time treatment. Although urine cytology and cystoscopy are still the standard of practice, many candidate biomarkers for bladder cancer are emerging [29]. Although several biomarkers (Oncoprotein DEK, survivin mRNA, Gc-globulin and IL-18 so on) for bladder cancer have been proposed [30–33], no single marker has emerged as the test of choice. Further research and better understanding of the biology of bladder cancer, improved diagnostic techniques, and standardized interpretation are essential steps to develop reliable biomarkers. From our results, Cx26 and KDM5B can be a novel combined adjuvant biomarker for bladder cancer diagnosis. The data suggest that a negative value to Cx26 immunohistochemical expression and a positive value to KDM5B immunohistochemical expression can be an ancillary diagnosis of primary bladder malignancy.

The inverse expression pattern between Cx26 and KDM5B was first reported here. Theoretically, there are many histone demethylases for methylated H3K4, the inhibition of Cx26 may be caused by other histone demethylases. However, KDM5B and Cx26 were proved to be monotonically related. We guess there are two reasons for explaining that. Firstly, a significant difference in expression levels is found only for the KDM5B between normal and cancer cells when JARID family are examined in a small subset of clinical bladder cancer samples [8]. Secondly, other histone demethylases may have the different functions with KDM5B although they both can remove the methyl group from demethylated H3K4. For example, LSD is not only a H3K4 demethylase but also a H3K9 demethylase [34], but KDM5B cannot be a H3K9 demethylase [6]. Our results suggest that histone epigenetics are closely related with connexins activities in the progression of bladder cancer. Much work needs to be done in the new field since many connexins and histone demethylases are revealed [35–37]. More potential connections between histone demethylases and connexins can be found with the work being taken forward. All the work has the benefit of understanding the mechanisms for the cause of bladder cancer.

## 3. Experimental Section

### 3.1. Cell Lines and Cell Culture

HT1376 and T24 bladder invasive transitional cell cancer cell lines (Watson Biotechnologies, Shanghai, China) were routinely grown in RPMI 1640 medium containing 10% of heat-inactivated FCS (Gibco BRL, Gaithersburg, MD, USA). Cells were incubated at 37 °C in a 50 mL/L CO_2_ air incubator with saturated humidity.

### 3.2. KDM5B Expression Constructs

KDM5B gene (Accession number: NM_006618.3) was amplified using the primers (Sense primer, 5′-GTGAGCTAGCATGGAGGCGGCCACCACACTGCACCC-3′; Antisense primer, 5′-CTGAGAA TTCTTACTTTCGGCTTGGTGCGTCCTTC-3′), generating a 4655-bp product. The PCR product was cloned into the *Nhe*I-*EcoR*I sites of pcDNA3.1 vector according to the manufacturer’s instructions (TOPO TA Expression Kit, Applied Biosystems China Limited, Beijing, China), which was named as pcDNA3.1-KDM5B. Plasmid pcDNA3.1-KDM5B was amplified in *E. coli*, isolated using QIAprep Miniprep Kit (QIAGEN China Co., Ltd., Shanghai, China) and verified by automated DNA sequencing.

### 3.3. ShRNA Constructs for KDM5B Gene Silencing

The pTZU6+1 expression plasmid was a gift from the Hepatitis Institute of Chongqing Medical University (Chongqing, China). According to shRNA design principles and the KDM5B coding sequence, 19–21 nt of the DNA oligos were designed such that they were spanned by TTCG insertion sequence with homology to the targeted gene. In this study, both the KDM5B coding sequence and the reverse complementary sequence were synthesized, named as follows: siKDM5B, sense 5′-TCGACCAGTGAATGAGCTCCGGCATTGGTGCCGGAGCTCATTCACTGTTTTTT-3′, antisense 5′-CTAGAAAAAACAGTGAATGAGCTCCGGCACCAATGCCGGAGCTCATTCACT GG-3′; siKDM5B#2, sense 5′-TCGACGGAATATGGAGCTGACATTTTGG AATGTCAGCTCCATATTCC TTTTTT-3′, antisense 5′-CTAGAAAAAAGGAATATGGAGCTGACATTCCAAAATGTCAGCTC CATATTCCG-3′. *Sal*I and *Xba*I restriction sites were incorporated on the either end of the oligos for the cloning into the pTZU 6+1 vectors. Thus, pTZU6+1-shRNA-KDM5B1 and pTZU6+1-shRNA-KDM5B2 vectors were constructed.

### 3.4. Transfection of T24 Cells, Western Blotting and Immunocytochemistry

The HT1376 and T24 cells (2 × 10^5^ per p-35 plate) were transfected with 2 μg of pcDNA3.1-KDM5B, pTZU6+1, pTZU6+1-shRNA-KDM5B1and pTZU6+1-shRNA-KDM5B2 as treatment groups. The HT1376 and T24 cells were transfected with 2 μg of pcDNA3.1 and pTZU6+1, as control groups. Transfection was performed in 50%–60% confluent cells in plates using 9 μL of Lipofectamine 2000TM (Applied Biosystems China Limited, Beijing, China). Forty-eight h after transfection, cells were split and were selected for neomycin resistant clones with 600 μg/mL G-418. Resistant colonies were either pooled or cloned by ring isolation.

KDM5B antibody and Cx26 antibody were purchased from Shengshizhongfang BioSci& Tech. Ltd. Co. (Beijing, China). Antibodies (anti-histone 3, anti-H3K4me3/2/1) for different histone methylations and β-actin antibody were purchased from Abcam (Beijing, China). Different tissues (normal bladder, bladder cancer from primary to metastatic tumors) were obtained from Southwest Hospital, Third Military Medical University (Chongqing, China) with prior approval from the Institutional Review Board. Three different samples were obtained from normal bladder (marked as “benign”, no tumor was found in the bladder), normal bladder cancer (marked as “cancer”, the tumor was found on or under the inner lining of the bladder; the tumor grew into the muscle layer of the bladder) and metastatic bladder cancer (marked as “metastatic”, the tumor grew through the bladder muscle into the fat layer surrounding the bladder; the tumor spread to surrounding organs, such as the prostate, bowel, vagina, or uterus). Bladder biopsies were collected via a cystoscope and stored under −80 °C. A total of 139 tissue pieces (*n* =38 for normal bladder, *n* = 46 for normal bladder cancer, and *n* = 55 for metastatic bladder cancer) were distinguished by a pathologist experienced with bladder cancer at our institution.

All the non-transfected and transfected cell lines, tumor and non-tumor tissues were homogenized in RIPA buffer (150 mM Sodium chloride, 1% NP-40, 0.5% sodium deoxycholate, 0.1% SDS, 50 mM Tris-HCl (pH 8.0)) and protease inhibitor CompleteMini (Roche R&D Center (China) Ltd., Shanghai, China) was included. After the debris was removed, supernatants were boiled and mixed with an equal volume of 20% glycerol containing 0.02% bromophenol blue. Proteins were separated by SDS–PAGE and transferred to a polyvinylidene difluoride membrane (Millipore, Billerica, MA, USA). The membranes were blocked with 5% skim milk in TBST (10 mM Tris (pH 7.5), 100 mM NaCl and 0.1% Tween-20) and incubated with primary antibodies in TBST with 0.5% skim milk overnight at 4 °C. The membrane was treated with primary antibodies and horseradish peroxidase-conjugated goat anti-mouse immunoglobulin G antibody (1:3000) (Amersham Biosciences, Piscataway, NJ, USA) as the secondary antibody. Immunoreactive bands were visualized by ECL (GE Healthcare, Shanghai, China) and quantified by densitometry with Image J software 1.45 (NIH, Bethesda, MD, USA) according to software’s instruction.

Bladder tissues were processed for immunohistochemistry immediately after removal. We performed immunohistochemistry for kit, Cx26 and KDM5B (Biocompare, South San Francisco, CA, USA). The relative immunohistochemical expression was quantitatively evaluated using NIH image program 1.57 computer software (NIH, Bethesda, MD, USA). A score of 0–300 was calculated for each case as the intensity of the product and the percent of relative immunohistochemical expression.

### 3.5. Reverse Transcription Polymerase Chain Reaction (RT-PCR) and Real Time qPCR

Cx26 and KDM5B expression levels were estimated using RT-PCR and real time qPCR. Real time qPCR was conducted using SYBR green I master mix (Light-Cycler 480, Roche R&D Center (China) Ltd., Shanghai, China). RNA was isolated from the non-transfected and transfected HT1376 and T24 cells, followed by cDNA synthesis and data analysis as described previously [7]. The primers for real time qPCR were: Cx26, 5′-TCTTCATTTTTCGCATTATG-3′ and 5′-CATGTCTCCGGTAGGCCA CG-3′; KDM5B, 5′-GAAGACCGGCTACTGTTGTG-3′ and 5′-AGCCAAATGCTTCTTGTGGC-3′; GAPDH, 5′-CCCTTCATTGACCTCAACTAC-3′ and 5′-CCACCTTCTTGATGTCATCAT-3′. GAPDH was used as an internal control.

### 3.6. Demethylation Reactions and MALDI-TOF Mass Spectrometry

The extracts of the non-transfected and transfected HT1376 and T24 cells were incubated with 10 mM of peptide (H3K4Me3/2/1) (H3 1–15): ART(Me3/2/1)KQTARKSTGGKA, molecular weights of 1601/1587/1573 for H3K4Me3/2/1 respectively) (synthesized by GL Biochem Ltd., Shanghai, China) or 5 mg of calf thymus type II-A histones (Sigma-Aldrich Trading Co, Ltd., Shanghai, China) in the DeMTase reaction buffer (20 mM Tris-HCl [pH 7.3], 150 mM NaCl, 50 mM (NH_4_)_2_Fe(SO_4_)_2_ + 6(H_2_O), 1 mM α-ketoglutarate, and 2 mM ascorbic acid) for 2 h at 37 °C. The reactions were inhibited by 10 mM EDTA and 250 mM DFO. One microliter of demethylation reaction mixture was desalted through a C18 ZipTip (Millipore, Billerica, MA, USA). The ZipTip was activated, equilibrated, and loaded. The bound material was then eluted with 10 mg/mL a-cyano-4-hydroxycinnamic acid MALDI matrix in 70% acetonitrile/0.1% TFA before being spotted and cocrystallized. The samples were analyzed by a MALDITOF/TOF mass spectrometer (Waters Corporation, Milford, MA, USA). The demethylating activity of KDM5B from normal bladder, normal bladder cancer and metastatic bladder cancer was determined using the method above mentioned.

### 3.7. Statistical Analysis

The association for the relative immunohistochemical expression between Cx26 and KDM5B in different groups was compared by one-way analysis of variance (ANOVA) followed by the post hoc test of Fisher’s protected least significant difference (LSD). We used Spearman’s rank correlation coefficient to identify the strength of correlation between Cx26 and KDM5B. The online software computes the Spearman Rank Correlation and the two-sided p-value. The ordinary scatterplot between ranks of X & Y is shown at: Spearman Rank Correlation—Free Statistics Software (Calculator) [38].

## 4. Conclusions

The inverse expression pattern between Cx26 and KDM5B is firstly reported here. Cx26 is down-regulated by KDM5B in the progression of bladder cancer. From the results, Cx26 and KDM5B can be a novel combined adjuvant biomarker for bladder cancer diagnosis. The data suggest that a negative value to Cx26 immunohistochemical expression and a positive value to KDM5B immunohistochemical expression can be an ancillary diagnosis of bladder malignancy.

## Supplementary Information
