# Supplementary material for: Connexin 26 is Down-Regulated by KDM5B in the Progression of Bladder Cancer
Source: Int J Mol Sci. 2013 Apr 11;14(4):7866–79. doi: 10.3390/ijms14047866 (PMC3645721; doi:10.3390/ijms14047866)
Supplement: Supplementary file 1 [file ijms-14-07866-s001.pdf]

Supplementary Information

Figure S1. The effects of KDM5B on the growth rate of HT1376 and T24 cells. The HT1376 and T24 cells transfected with pcDNA3.1-KDM5B, pTZU6+1-shRNA-KDM5B1 and pTZU6+1-shRNA-KDM5B2 were regarded as treatment groups. The HT1376 and T24 cells were regarded as control groups. All cells (2×10^5 per p-35 plate) were routinely grown in RPMI 1640 medium and incubated at 37 °C in a 50 mL/L CO_2 air incubator with saturated humidity. (**A**) The *in vitro* tests showed the inhibition of growth rate of the HT1376 and T24 cells transfected with pcDNA3.1-KDM5B comparing with corresponding non-transfected cell lines; (**B**) the *in vitro* tests showed the increase of growth rate of the HT1376 and T24 cells transfected with pTZU6+1-shRNA-KDM5B1 and pTZU6+1-shRNA-KDM5B2 comparing with corresponding non-transfected cell lines. Each bar represented the mean ± S.D. of three independent experiments.

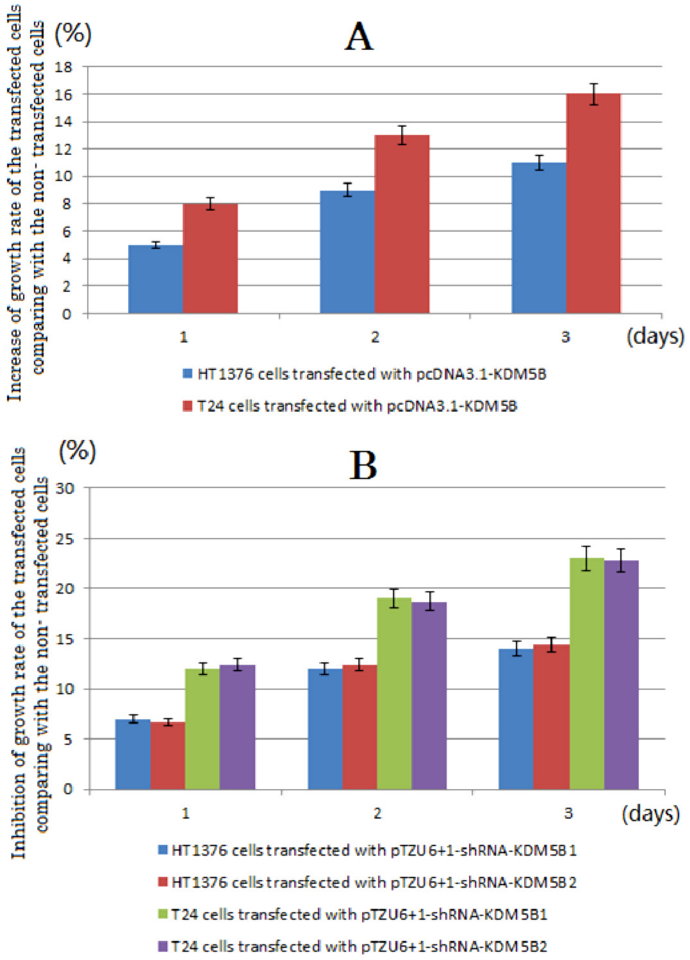

© 2013 by the authors; licensee MDPI, Basel, Switzerland. This article is an open access article distributed under the terms and conditions of the Creative Commons Attribution license (http://creativecommons.org/licenses/by/3.0/).
